# Initial and supplementary indication approval of new targeted cancer drugs by the FDA, EMA, Health Canada, and TGA

**DOI:** 10.1007/s10637-022-01227-5

**Published:** 2022-04-07

**Authors:** Daniel Tobias Michaeli, Mackenzie Mills, Thomas Michaeli, Aurelio Miracolo, Panos Kanavos

**Affiliations:** 1 Fifth Department of Medicine, University Hospital Mannheim, Heidelberg University, Mannheim, Germany; 2 Department of Personalized Oncology, University Hospital Mannheim, Heidelberg University, Mannheim, Germany; 3 Department of Health Policy and Medical Technology Research Group – LSE Health, London School of Economics and Political Science, London, UK; 4 Division of Personalized Medical Oncology, German Cancer Research Center (DKFZ), Heidelberg, Germany

**Keywords:** Cancer drugs, Targeted therapy, Orphan, Accelerated approval, Drug development, Clinical trial, Drug pricing, Pharmaceutical policy, Indication-specific pricing

## Abstract

*Background*. Previous research focused on the clinical evidence supporting new cancer drugs’ initial US Food and Drug Administration (FDA) approval. However, targeted drugs are increasingly approved for supplementary indications of unknown evidence and benefit. *Objectives*. To examine the clinical trial evidence supporting new targeted cancer drugs’ initial and supplementary indication approval in the US, EU, Canada, and Australia. *Data and Methods*. 25 cancer drugs across 100 indications were identified with FDA approval between 2009–2019. Data on regulatory approval and clinical trials were extracted from the FDA, European Medicines Agency (EMA), Health Canada (HC), Australian Therapeutic Goods Administration (TGA), and clinicaltrials.gov. Regional variations were compared with χ^2^-tests. Multivariate logistic regressions compared characteristics of initial and supplementary indication approvals, reporting adjusted odds ratios (AOR) with 95% confidence intervals (CI). *Results*. Out of 100 considered cancer indications, the FDA approved 96, the EMA 92, HC 86, and the TGA 83 (83%, p < 0.05). The FDA more frequently granted priority review, conditional approval, and orphan designations than other agencies. Initial approvals were more likely to receive conditional / accelerated approval (AOR: 2.69, 95%CI [1.07–6.77], p < 0.05), an orphan designation (AOR: 3.32, 95%CI [1.38–8.00], p < 0.01), be under priority review (AOR: 2.60, 95%CI [1.17–5.78], p < 0.05), and be monotherapies (AOR: 5.91, 95%CI [1.14–30.65], p < 0.05) than supplementary indications. Initial indications’ pivotal trials tended to be shorter (AOR per month: 0.96, 95%CI [0.93–0.99], p < 0.05), of lower phase design (AOR per clinical phase: 0.28, 95%CI [0.09–0.85], p < 0.05), and enroll more patients (AOR per 100 patients: 1.19, 95%CI [1.01–1.39], p < 0.05). *Conclusions*. Targeted cancer drugs are increasingly approved for multiple indications of varying clinical benefit. Drugs are first approved as monotherapies in rare diseases with a high unmet need. Whilst expedited regulatory review incentivizes this prioritization, indication-specific safety, efficacy, and pricing policies are necessary to reflect each indication’s differential clinical and economic value.

**Supplementary Information:**

The online version contains supplementary material available at 10.1007/s10637-022-01227-5.

## Introduction

Oncology drugs are increasingly approved for multiple solid and hematologic cancer entities. Especially, immune and gene therapies, which target specific disease pathways shared by many malignancies, are effective across multiple indications. Consequently, after the initial US Food and Drug Administration (FDA) approval, cancer drugs are often approved for various supplementary indications. This is especially relevant, given that supplementary indications accounted for 75% of oncology approvals in 2018 [1]. However, the inherent clinical benefit of such drugs may vary across indications and patient groups [2]. Marketing authorization (MA) and health technology assessment (HTA) agencies ought to account for the differential pharmacological and economic characteristics of multi-indication drugs. Regulatory approval must particularly address fluctuating safety and efficacy characteristics of indications.

### Regulatory approval of cancer drugs

In this context, regulatory agencies can employ expedited review pathways to prioritize clinical development and approval of indications targeting diseases with a high unmet clinical need for a small population group. The FDA currently employs several approval pathways including fast track, priority review, and breakthrough therapy which not only speed up clinical and regulatory timelines, but also offer procedural support to pharmaceutical companies [3]. Indications which “treat serious conditions, and […] fill an unmet medical need based on a surrogate endpoint” may be granted accelerated (FDA) or conditional approval (EMA) [4, 5]. Additionally, regulatory agencies may prioritize the development of drugs treating rare diseases or drugs that would otherwise not be financially viable for pharmaceutical companies to develop by granting orphan designation status [6]. “Orphan drugs are medicines or vaccines intended to treat, prevent or diagnose a rare disease” [7]. Whilst “the definition of rare diseases varies across jurisdictions [it] typically considers disease prevalence, severity and existence of alternative therapeutic options” [7].

### Economics of targeted multi-indication cancer drugs

Regulatory agencies utilize these expedited review processes on an indication-specific level to prioritize drug-indications which are believed to deliver a high clinical benefit to patients [8]. Similarly, pharmaceutical companies are incentivized to pursue research and development (R&D) in diseases with a high unmet therapeutic need with expedited clinical development and regulatory approval timelines [9]. Michaeli et al. previously demonstrated this strategy yields high company valuations and excess financial returns for Bioentrepreneurs and Investors [10, 11]. Arguably more substantial than R&D incentives could be the monetary incentive created by uniform pricing of multi-indication drugs. Uniform pricing sets one list price per drug. Therefore, pharmaceutical companies would theoretically want to sequence indication approval in order to extract the highest possible price and profit [12, 13]. Consequently, primary launching drugs in indications with strong clinical benefit and high value to a small patient group allows companies to establish an initial high list price.

### Objectives

Both indication prioritization by regulators and deliberate indication launch sequencing by pharmaceutical companies could influence the timelines and characteristics of newly approved multi-indication drugs. Previous research examined the economic theory of multi-indication drugs [12, 14, 15], discussed pricing strategies [, 13, 16–21], conducted indication-specific healthcare evaluations [2, 22–24], and explored case studies of single multi-indication drugs in a distinct country [24, 25]. Current evidence investigating regulatory approval of multi-indication drugs on a global scale is missing. Whilst previous research analyzed the benefit of initial cancer drug approvals [26, 27], little is known about the clinical trial evidence supporting the approval of supplementary indications. This paper examines the effects of clinical and regulatory processes on multi-indication drug approvals across four jurisdictions: The US, Europe, Canada, and Australia. The objective is to, first, compare and contrast approval of targeted cancer drugs across regulatory agencies. Thereafter, initial and supplementary indications are compared regarding their regulatory approval, clinical trial evidence, and treatment characteristics in multivariate logistic regression models.

## Data and methods

### Taxonomy of targeted multi-indication cancer drugs

Given the existence of different multi-indication drug types, a methodological taxonomy was created to classify multi-indication drugs into three groups according to indication similarity [12, 21, 25]:

#### Indications across distinct therapeutic areas

The first and broadest category entails drugs with indications across distinct therapeutic areas. For instance, anti-VEGF inhibitor aflibercept treats colorectal cancer (oncology) and macular degeneration (ophthalmology).

#### Indications across different disease areas

The second group only considers drugs within the same therapeutic area, but across different disease areas. For example, cabozantinib is used in oncology to treat hepatocellular, thyroid, and advanced renal cancer.

#### Indications across different lines of therapy

The third and narrowest category only includes drugs within the same therapeutic and disease area, but across different lines of therapy. Afatinib was first approved for 2nd line advanced or metastatic non-small cell lung cancer and then extended for the same disease as 1st line treatment.

### Data

#### Sample selection

**Fig. 1:**
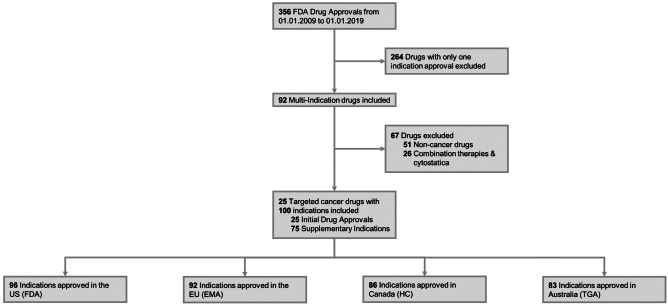
Flow Diagram of New Targeted Multi-Indication Cancer Drugs Included in the Analysis, 2009–2019 FDA: US Food and Drug Administration, EMA: European Medicines Agency, HC: Health Canada, TGA: Therapeutics Goods Administration

356 FDA drug approvals from 01.01.2009 to 01.01.2019 were screened to identify 92 drugs with multiple indications (Fig. 1). 25 of these multi-indication drugs were selected for further analyses. Selection was based on four prioritization criteria: first, it included drugs from all three drug groups (defined in Taxonomy of Targeted Multi-Indication Cancer Drugs); second, it focused on oncology drugs in the first approved indication to ensure comparability of clinical trial data; third, it prioritized indications with at least two monotherapy treatments in order to exclude confounding effects of combination treatments; and finally, it concentrated on targeted therapeutics.

#### Data sources

Data for the selected 25 targeted multi-indication cancer drugs were collected from four regulatory agencies: US Food and Drug Administration (FDA), European Medicines Agency (EMA), Health Canada (HC), and Therapeutic Goods Administration (TGA). Public availability of regulatory documents (MA reports) and language (English, French) were the main criteria for country selection. For all 25 drugs, MA data were collected from the respective agency website (Supplementary Table e1). Regulatory approval reports were matched with pivotal trial data found on clinicaltrials.gov.

#### Collected variables

Several variables collected from MA reports were considered relevant to assess the launch sequencing of indications according to their value, disease prevalence, and clinical evidence. Evidence may vary in quality, e.g. clinical trial design, endpoints, outcome, and quantity, e.g. additional supporting trials. Regulatory agencies may approve indications under expedited pathways, offer special review assistance, and grant prolonged exclusivity periods [3].

The following variables were extracted from MA reports:

 1) molecule name, 2) brand name, 3) therapeutic indication, 4) marketing authorisation date, 5) marketing authorisation type (standard approval, conditional approval, priority review), 6) orphan designation, and 7) additional supporting clinical trials. “Regular approval” (EMA, FDA, TGA) and “Notice of Compliance” (HC) were classified as standard approval. Conditional approvals were identified as “Conditional marketing authorisation or Marketing Authorisation under exceptional circumstances” (EMA), “Accelerated Approval” (FDA), “Notice of Compliance with Conditions” (HC), and “Provisional Approval” (TGA). “Priority review” (FDA, HC, TGA) and “Accelerated Approval” (EMA) were categorized as priority review.

Pivotal trial data found in MA reports were matched with clinical trial characteristics found on clinicaltrials.gov. Variables on 1) trial design, 2) number of enrolled patients, 3) primary endpoint type, 4) primary endpoint outcome, 5) trial start date, and 6) primary completion date were collected.

### Statistical analysis

Following data extraction and storage in Microsoft EXCEL, analyses were performed with STATA SE Version 15.1. First, baseline characteristics of multi-indication cancer drug approvals were examined. Thereafter, approvals were compared across regions with χ^2^-tests. Maximum-likelihood logistic regression models were constructed to assess the association of collected variables with indication approval sequence. The dependent variable $({Y}_{1/0})$ was coded as “1” for initial and “0” for supplementary indications according to FDA approval date:

1 $${Y}_{1/0}\begin{cases}Y=1, &if\ initial\ drug\ approval\ \\ Y=0, &if\ supplementary\ indication\ approval\end{cases}$$

Independent variables $({x}_{i})$ included orphan status, treatment type, marketing authorization type, treatment line, supporting clinical trials, pivotal trial design, length, endpoint, and outcome. Clinical trial performance was translated to the European Society for Medical Oncology's modified Magnitude of Clinical Benefit Scale (MCBS). The models accounted for country characteristics, clustering of observations, and heteroskedastic standard errors. First, the association of variables with indication approval order was explored in univariate regression models:

2 $$Logit\left({Y}_{1/0} \right|{ X}_{1}={x}_{1}) ={\beta }_{0}+{x}_{1}{\beta }_{1}$$

Thereafter, a multivariate logistic regression model was constructed with all previously identified variables:

3 $$Logit\left({Y}_{1/0} \right|{ X}_{i}={x}_{i}) ={\beta}_{0}+{x}_{i1}{\beta}_{1}+{x}_{i2}{\beta}_{2}+...+{x}_{ik}{\beta }_{k}$$

Odds ratios (OR), adjusted odds ratios (AOR), and 95% confidence intervals (CI) of the coefficients $({\beta}_{k})$ and p-values are presented:

4 $$Odds\left({Y}_{1/0} \right|{ X}_{i}={x}_{i}) ={\mathrm{exp}(\beta}_{0}+{x}_{i1}{\beta }_{1}+{x}_{i2}{\beta}_{2}+...+{x}_{ik}{\beta}_{k})$$

## Results

25 targeted cancer drugs across 100 indications were analyzed (Table 1 and Supplementary Table e2). Consequently, 25 indications of the 100 screened indications were categorized as “initial indication” and 75 as “supplementary indication”, based on their FDA approval date. The majority of indications were monotherapy (82.0%) and 2nd, 3rd, or 4th line (62.0%) treatments. Data were collected on indications across therapeutic areas (20.0%), across disease areas (57.0%), and across different lines of therapy (23.0%). The number of indication approvals varied by regulatory agency: FDA (96.0%), EMA (92.0%), HC (86.0%), and TGA (83.0%) (χ^2^_(3, N=400)_ = 10.71, p < 0.05). The aggregate sample size consequently consists of 357 regulatory approvals.

**Table 1:** Baseline Sample Characteristics of FDA Approved Targeted Multi-Indication Cancer Drugs

	**No**	**(%)**
FDA Approval Sequence		
Initial Indication	25	(25.0%)
Supplementary Indication	75	(75.0%)
Line of Treatment		
1st line	38	(38.0%)
2nd, 3rd, 4th line	62	(62.0%)
Treatment Type		
Monotherapy	82	(82.0%)
Combination	18	(18.0%)
Multi-Indication Drug Type		
Across Therapeutic Areas	20	(20.0%)
Across Disease Areas	57	(57.0%)
Across Lines of Therapy	23	(23.0%)
**Total No. of Indications**	**100**	**(100%)**

*FDA* US Food and Drug Administration, *EMA* European Medicines Agency, *HC* Health Canada, *TGA* Therapeutics Goods Administration

In the US, all drugs were considered for only one indication during initial approval. In contrast, companies more frequently applied directly for multiple indications during the initial approval in other jurisdictions. For instance, tisagenlecleucel’s initial drug approval by the EMA, HC, and TGA entailed two indications. Atezolizumab and imbruvica were simultaneously approved for two indications by the EMA and TGA.

### Comparing regulatory approvals across agencies

The considered indications were more likely to receive a conditional approval from the FDA (29.2%) and HC (26.7%) compared to the EMA (13.1%) and TGA (3.6%) (χ^2^_(6, N=357)_ = 47.90, p <0.001). Similar findings were observed for the priority review status (FDA: 16.6%, HC: 25.6%, EMA: 6.5%, TGA: 9.6%). Approved indications were also more likely to receive orphan designation status from the FDA (49.0%) than other agencies (EMA: 23.9%, TGA: 9.6%) (χ^2^_(3, N=357)_ = 35.62, p <0.001). Differences were also observed for supporting clinical trials submitted alongside the pivotal trial (χ^2^_(9, N=357)_ = 34.35, p <0.001). Additional evidence was not available in more than half of the reports issued by the FDA, HC, and TGA (Table 2). However, 72.8% of the EMA reports entailed additional clinical trial evidence. No difference could be observed between pivotal trial and treatment characteristics of approved indications across settings (Supplementary Table e3).

**Table 2:** Regulatory Approval Characteristics of Targeted Multi-Indication Drugs Across the US (FDA), EU (EMA), Canada (HC), and Australia (TGA)

	**US** **(FDA)**		**EU** **(EMA)**		**Canada** **(HC)**		**Australia** **(TGA)**		*P-value*
	**No**	**(%)**	**No**	**(%)**	**No**	**(%)**	**No**	**(%)**	
Approval Type									< *.001*
Standard	52	(54.2%)	74	(80.4%)	41	(47.7%)	72	(86.8%)	
Conditional Approval	28	(29.2%)	12	(13.1%)	23	(26.7%)	3	(3.6%)	
Priority Review	16	(16.6%)	6	(6.5%)	22	(25.6%)	8	(9.6%)	
Orphan Designation									< *.001*
No	49	(51.0%)	70	(76.1%)	NA	NA	75	(90.4%)	
Yes	47	(49.0%)	22	(23.9%)	NA	NA	8	(9.6%)	
MA Supporting Trial ^†^									< *.001*
No	57	(59.4%)	25	(27.2%)	48	(55.8%)	50	(60.2%)	
Phase 1	7	(7.3%)	14	(15.2%)	5	(5.8%)	2	(2.4%)	
Phase 2	15	(15.6%)	28	(30.4%)	13	(15.1%)	19	(22.9%)	
Phase 3	17	(17.7%)	25	(27.2%)	20	(23.3%)	12	(14.5%)	
Pivotal Trial Design ^‡^									*0.822*
Phase 1	4	(4.2%)	3	(3.2%)	5	(5.8%)	3	(3.6%)	
Phase 2	25	(26.0%)	18	(19.6%)	23	(26.8%)	19	(21.7%)	
Phase 3	67	(70.8%)	71	(77.2%)	58	(67.4%)	61	(74.7%)	
**No. of Observations**	**96**	**(100%)**	**92**	**(100%)**	**86**	**(100%)**	**83**	**(100%)**	

P-values calculated based on χ^2^-tests

* FDA *US Food and Drug Administration, *EMA* European Medicines Agency, *HC* Health Canada, *TGA* Therapeutics Goods Administration, *NA* Not Applicable

^†^ Highest phase of *supporting* trials disclosed in the regulatory approval report (No supporting trial: 0, Phase 1: 1, Phase 2: 2, Phase 3: 3)

^‡^ Highest phase *pivotal* trial disclosed in the regulatory approval report (Phase 1: 1, Phase 2: 2, Phase 3: 3)

### Comparing initial and supplementary indication characteristics

Initial and supplementary indication approvals were compared regarding their regulatory (MA type, orphan designation, supporting trials), pivotal trial (trial design, primary endpoint type, MCBS, enrolled patients, trial length), and treatment characteristics (treatment line, treatment type, indication group). Results of the univariate analysis are displayed in Table 3.

**Table 3:** Univariate Comparison between Initial and Supplementary Indication Approval of New Targeted Multi-Indication Cancer Drugs by the FDA, EMA, HC, and TGA

	**Initial** **Indication**		**Supplementary** **Indication**		**Univariate**		
	**No**	**(%)**	**No**	**(%)**	**OR**	**[95% CI]**	***P-value***
**A) Regulatory Approval**							
Approval Type							
Standard	48	(50.0%)	191	(73.2%)	1.00	[Reference]	
Conditional Approval	29	(30.2%)	37	(14.2%)	3.89	[1.65–9.17]	*0.002*
Priority Review	19	(19.8%)	33	(12.6%)	2.75	[1.25–6.04]	*0.012*
Orphan Designation							
No	54	(56.2%)	203	(77.8%)	1.00	[Reference]	
Yes	42	(43.8%)	58	(22.2%)	2.72	[1.33–5.57]	*0.006*
MA Supporting Trial ^†^							
No	30	(31.2%)	150	(57.4%)	1.00	[Reference]	
Phase 1	4	(4.2%)	24	(9.2%)	0.92	[0.30–2.78]	*0.882*
Phase 2	31	(32.3%)	44	(16.9%)	3.75	[1.62–8.70]	*0.002*
Phase 3	31	(32.3%)	43	(16.5%)	3.83	[1.54–9.52]	*0.004*
**B) Pivotal Trial Characteristics**							
Trial Design ^‡^							
Phase 1	8	(8.3%)	7	(2.7%)	1.00	[Reference]	
Phase 2	31	(32.3%)	54	(20.7%)	0.50	[0.07–3.66]	*0.495*
Phase 3	57	(59.4%)	200	(76.6%)	0.25	[0.04–1.59]	*0.140*
Primary Endpoint							
Surrogate	70	(72.9%)	167	(64.0%)	1.00	[Reference]	
Clinical	20	(20.8%)	43	(16.5%)	1.11	[0.36–3.44]	*0.859*
Co-Primary	6	(6.3%)	51	(19.5%)	0.28	[0.06–1.34]	*0.112*
MCBS Score							
Score of 1, 2, or 3	40	(41.7%)	159	(60.9%)	1.00	[Reference]	
Score of 4 or 5	56	(58.3%)	102	(39.1%)	2.20	[0.94–5.16]	*0.070*
Enrolled Patients (per 100)							
Mean [95% CI]	547	[465–629]	585	[533–637]	0.98	[0.88–1.09]	*0.687*
Trial Length (months)							
Mean [95% CI]	29	[26–32]	34	[31–36]	0.98	[0.95–1.01]	*0.189*
**C) Treatment Characteristics**							
Treatment Type							
Combination	4	(4.2%)	55	(21.1%)	1.00	[Reference]	
Monotherapy	92	(95.8%)	206	(78.9%)	6.17	[1.20–31.8]	*0.030*
Line of Treatment							
2nd, 3rd, or 4th Line	68	(70.8%)	153	(58.6%)	1.00	[Reference]	
1st Line	28	(29.2%)	108	(41.4%)	0.58	[0.22–1.55]	*0.279*
Multi-Indication Drug Type							
Across Therapeutic Areas	20	(20.8%)	50	(19.2%)	1.00	[Reference]	
Across Disease Areas	38	(39.6%)	160	(61.3%)	0.59	[0.17–2.04]	*0.409*
Across Lines of Therapy	38	(39.6%)	51	(19.5%)	1.86	[0.50–6.97]	*0.355*

Indication approval sequence was determined by the FDA approval date (initial indication: 1, subsequent indication: 0). MCBS: Magnitude of Clinical Benefit Scale (1: *low* benefit to 5: *high* benefit)

^†^ Highest phase of *supporting* trials disclosed in the regulatory approval report

^‡^ Highest phase *pivotal* trial disclosed in the regulatory approval report

All regulatory approval characteristics showed significant differences across indication approval sequence. However, clinical trial characteristics did not reveal significant discrepancies. Variables describing treatment characteristics offer mixed insights.

### Multivariate regression comparing initial and supplementary indication approvals

Results of the univariate and correlation analysis informed the construction of the multivariate regression model. The multivariate regression was constructed in a stepwise manner to recognize the impact of variable categories on indication approval order. Therefore, regulatory (Model 1), pivotal trial (Model 2), and treatment (Model 3) characteristics were added sequentially. The final Model 4 optimizes the regression by excluding multi-collinear variables. Results of the multivariate regressions are presented in Table 4.

**Table 4:** Logistic Regression Comparing Initial and Supplementary Indication Approval of New Targeted Multi-Indication Cancer Drugs by the FDA, EMA, HC, and TGA

	**Model 1**	**Model 2**	**Model 3**	**Model 4**
*Dependent Variable: FDA Approval Sequence (Initial Indication: 1, Supplementary Indication: 0)*				
**A) Regulatory Approval**				
Conditional Approval	3.208*	2.168	2.684*	2.686*
	(2.46)	(1.70)	(2.06)	(2.09)
Priority Review	2.663*	2.606*	2.482*	2.602*
	(2.38)	(2.50)	(2.27)	(2.35)
Orphan Designation	2.807*	3.703**	3.689**	3.318**
	(2.29)	(2.85)	(2.87)	(2.67)
Supporting Trials ^†^	1.704***	1.712***	1.703**	1.654**
	(3.50)	(3.41)	(3.15)	(3.02)
**B) Pivotal Trial Characteristics**				
Phase ^‡^		0.230*	0.206*	0.281*
		(-2.43)	(-2.55)	(-2.25)
MCBS Score		0.853	0.607	
		(-0.27)	(-0.81)	
Enrolled Patients (per 100)		1.153	1.195*	1.186*
		(1.85)	(2.15)	(2.14)
Trial Length (months)		0.963*	0.961*	0.963*
		(-2.18)	(-2.31)	(-2.28)
**C) Treatment Characteristics**				
Monotherapy			7.174*	5.913*
			(2.25)	(2.12)
1st Line Treatment			1.318	1.179
			(0.48)	(0.30)
**Agency Dummy**				
US (FDA)	1.000	1.000	1.000	1.000
	(Reference)	(Reference)	(Reference)	(Reference)
EU (EMA)	1.366	1.339	1.389	1.382
	(1.37)	(1.21)	(1.31)	(1.34)
Canada (HC)	1.180	1.257	1.209	1.155
	(0.76)	(0.96)	(0.78)	(0.62)
Australia (TGA)	2.711**	2.828***	3.000***	2.891***
	(3.20)	(3.27)	(3.29)	(3.29)
No. of Observations	357	357	357	357
Pseudo-R^2^	13.9%	19.7%	22.8%	22.4%
AIC	374	358	349	349
Wald-Test (p-Value)	0.0001	0.0003	0.0001	0.0001

Regulatory (Model 1), pivotal trial (Model 2), and treatment (Model 3) characteristics were added sequentially. Model 4 optimizes the regression by excluding collinear variables. Odds Ratios are presented. Indication approval sequence was determined by the FDA approval date (initial indication: 1, supplementary indication: 0). t statistics in parentheses

*FDA* US Food and Drug Administration, *EMA* European Medicines Agency, *HC* Health Canada, *TGA* Therapeutics Goods Administration, *MCBS* Magnitude of Clinical Benefit Scale (1: *low* benefit to 5: *high* benefit)

P-values: * *p* <*0.05*; ** *p* <*0.01*; *** *p* <*0.001*

^†^ Highest phase of *supporting* trials disclosed in the regulatory approval report (No supporting trial: 0, Phase 1: 1, Phase 2: 2, Phase 3: 3)

^‡^ Highest phase *pivotal* trial disclosed in the regulatory approval report (Phase 1: 1, Phase 2: 2, Phase 3: 3)

#### Regulatory approval characteristics

Initial indications were significantly more likely to receive conditional approval, orphan designation and be under priority review compared to supplementary indications. Conditional approval was received by 30.2% of initial and 14.2% of supplementary indications (AOR: 2.69, 95%CI [1.07–6.77], p <0.05). Priority review was granted to 19.8% of initial and 12.6% of supplementary indications (AOR: 2.60, 95%CI [1.17–5.78], p <0.05). Orphan designation status was granted to 43.8% of initial relative to 22.2% of supplementary indications (AOR: 3.32, 95%CI [1.38–8.00], p <0.01).

Significantly more later-stage supporting clinical trials were disclosed for the approval of initial indications compared to extensions. Compared to no supporting clinical trial, Phase 2 and Phase 3 supporting trials were significantly more frequent in initial regulatory approvals (AOR: 1.65, 95%CI [1.19–2.29], p <0.01).

#### Clinical trial evidence and benefit

Pivotal trials of initial approvals tend to be shorter, of lower phase design, and enroll more patients. Pivotal trials were of lower phase design for initial relative to supplementary indications (AOR per clinical development phase: 0.28, 95%CI [0.09–0.85], p < 0.05). Regulatory agencies granted approval to initial indications more frequently based on the clinical evidence provided by Phase 1 or 2 trials (40.6%), whereas supplementary indications mostly received approval after Phase 3 trials (76.6%). Accordingly, the average pivotal trial length amounted to 29 months (95%CI [26–32]) for initial and 34 months (95%CI [31–36]) for supplementary indications (AOR per month: 0.96, 95%CI [0.93–0.99], p <0.05).

Analysis of the number of enrolled patients is more complex. Simply comparing the mean number of patients might imply that initial approvals’ pivotal trials are of smaller size – initial indication: 547 (95%CI [465–629]), supplementary indication: 584 (95%CI [533–627]). However, after adjusting for orphan designation as well as pivotal trial phase, the opposite conclusion can be drawn: initial approvals enroll more patients (AOR per 100 patients: 1.19, 95%CI [1.01–1.39], p <0.05). Adjusting for orphan designation essentially captures disparities in disease prevalence, which is crucial for the design of clinical trials given that rare disease therapeutics are commonly tested on a smaller sample size of patients. Accordingly, Phase 2 trials, which are more frequently used for initial indications, register less patients than Phase 3 trials, which are the more frequent trial design for supplementary indications. In summary, initial indications pivotal trials enroll more patients on average, even though they are based on shorter Phase 1/2 designs. This observation only becomes apparent after adjusting for disease prevalence, as measured by the orphan designation variable, given that initial indications are more frequently approved for orphan disease.

A high clinical benefit, measured by a MCBS score of 4 or 5, was more frequently observed for initial (58.3%) than supplementary (39.1%) indications (OR: 2.20, 95%CI [0.94–5.16] p = 0.070). However, this observation was not significant in the multivariate model given that the MCBS score is highly correlated to the pivotal trial phase and endpoint as well as conditional, priority, and orphan designation status (Supplementary Table e4).

#### Treatment characteristics

Treatment characteristics showed significant differences for treatment types, but not for treatment line. Monotherapy treatment was more prevalent in the initial (95.8%) compared to the supplementary (78.9%) approval (AOR: 5.91, 95%CI [1.14–30.65], p <0.05). Indications in 1st line treatments were less frequently noticed for the initial (29.2%) relative to supplementary (41.4%) approval (OR: 0.58, 95%CI [0.22–1.55], p = 0.279).

#### Further consideration

Overall, Pseudo-R^2^ values suggest that regulatory characteristics explain 13.9% of the variation in indication approval order. Pivotal trial characteristics increased the Pseudo-R^2^ by an additional 5.8% whereas the treatment type marginally increased the Pseudo-R^2^ by 3.1%. Nevertheless, all included variables provide additional statistically significant benefit to the overall model fit as displayed by the decreasing AIC score. Additionally, model specification was ensured by conducting the link test as proposed by Pregibon [28].

## Discussion

With the rise of targeted, gene, hormone, and immuno-oncology therapies, oncology drugs are increasingly developed to treat multiple cancer types for specific patient groups. Regulatory agencies must consequently adapt their review pathways to ensure that safety and efficacy requirements are met across all indication approvals. Therefore, this paper first compared and contrasted the regulatory approval of 25 drugs with 100 indications across regulatory agencies in the US (FDA), EU (EMA), Canada (HC), and Australia (TGA). Thereafter, initial and supplementary indication approvals were compared regarding clinical trial evidence and treatment characteristics in a series of multivariate logistic regressions.

### Approval Pathways Across Agencies

#### Conditional approval and priority review

While all examined agencies employ special approval pathways to accelerate clinical development and marketing authorization, the conditions and use of such pathways vary significantly. Priority review status was received by 16.6% of FDA, 6.5% of EMA, 25.6% of HC, and 9.6% of TGA approved indications. The regulatory review process can thereby be expedited by up to four months – FDA/HC: 120, TGA: 90, EMA: 60 days [29–32]. All agencies commonly agree that priority review is granted to drugs significantly improving “the safety or effectiveness of the treatment, diagnosis, or prevention of serious conditions” relative to the standard of care [30]. However, no universal strict rules exist specifying precise requirements for this status. Therefore, safety and efficacy improvements have to be judged on a local level, ultimately resulting in regional discrepancies in priority review designations. Similar observations were apparent for conditional approvals across jurisdictions. Conditional approval permits for the “earlier approval of drugs that treat serious conditions, and that fill an unmet medical need based on a surrogate endpoint” [4]. Therefore, conditional approvals frequently permit the early authorization of indications only based on clinical evidence from Phase 1 or 2 trials.

#### Orphan designation

The contrasted agencies differentially utilize the orphan designation status. The status is more frequently granted by the FDA (49.0%), EMA (23.9%), and TGA (9.6%) while no such designation exists from HC. These variations are consistent with previous literature [33, 34]. Prevalence requirements are currently homogenous across the FDA, EMA, and TGA. The US Orphan Drug Act 1983 (ODA) states that not more than 200,000 patients may be affected by an orphan disease, which approximately translates into the prevalence threshold of 5 in 10,000 citizens defined in Europe (Orphan Drug Regulation 141/2000) and Australia (Therapeutic Goods Regulations 16 J/1997). However, the low rate of orphan designations in Australia might be explained by the former primary criterion that limits the status to a maximum of 2,000 suffering patients (approx. 1 in 10,000 citizens) [35]. Consequently, the TGA revised their orphan drug designation eligibility criteria in 2018 [36]. Additionally, the TGA only grants orphan status to one indication per drug. Moreover, the occurrence of rare, especially genetic, diseases varies between regions [37].

FDA and EMA commonly grant companies developing drugs for orphan disease several benefits. Advantages include research grants, fee reductions, expedited reviews, administrative assistance, and most importantly a period of market exclusivity [38, 39]. Market exclusivity restricts competition for a period of seven (FDA) or ten years (EMA). In contrast, TGA’s orphan designation only grants a fee reduction and an implicit five-year market exclusivity period. These weak financial incentives combined with a relatively small population of 25 million may cause pharmaceutical companies to be financially reluctant to file for regulatory approval in Australia.

#### Additional clinical evidence

Varying amounts of supporting evidence is disclosed by agencies in their approval reports. Arguably this observation could be due to two explanations. Either pharmaceutical companies submitted varying amounts of supporting evidence to agencies or agencies consider less of the same supporting evidence eligible for the examined indication.

### Comparing initial and supplementary indication approvals

In theory, single pricing policies incentivize pharmaceutical companies to deliberately sequence the development of indications according to clinical benefit and disease prevalence in order to maximize drug prices [12]. Similarly, conditional marketing authorization, expedited regulatory approval, and orphan designations theoretically prioritize indications targeting serious diseases with a high unmet clinical need [40, 41]. While our results provide evidence for both hypotheses it remains questionable which one is the driving factor. The econometric model finds that drugs are first approved as monotherapies in indications with a low disease prevalence, demonstrated by the orphan status, with a high potential clinical benefit, exhibited by the priority review status. Ultimately, evidence for initial indications is convincing enough to more frequently grant approval on well-designed large Phase 2 trials.

Initial indications were more frequently granted orphan designation status, thereby gaining all advantages of orphan drugs. A period of marketing exclusivity and limited negotiation power of payers and patients in rare disease areas with a large unmet need enable pharmaceutical companies to maximize orphan drug prices [42, 43]. While pharmaceutical companies argue for substantial R&D costs of these first indications [43], subsequent launches for high prevalence diseases may dynamically offset initial R&D expenses. Orphan drugs are not only assessed based on regular cost-effectiveness methods, but other dimensions of benefit, known as social value judgments, may apply [44]. Therefore, the consideration of additional dimensions of value can lead to the reimbursement of cost-ineffective drugs with high prices and limited established efficacy [42, 45].

Initial indications were significantly more likely to receive a conditional approval and be granted expedited regulatory reviews. Both designations are only granted to drugs that offer a significant efficacy or safety improvement relative to the standard of care in areas of high unmet clinical need. Consequently, results imply that initial approvals could provide a higher clinical benefit for patients than supplementary indications. Similarly, it was observed that initial indications more frequently had a high clinical benefit, measured by a MCBS score of 4 or 5, than supplementary indications. However, this observation was not significant in the multivariate regression due to collinearity with other variables that are associated with a drug’s clinical benefit. Even though the MCBS is a widely used and validated tool to rank and compare clinical endpoint performance [46], it does not reflect quality of life considerations [47]. More dimensional measures, such as quality-adjusted life years gained, are necessary to quantify and compare the clinical benefit of indications across therapeutic areas.

Pivotal trial design and endpoints significantly differed across the approval of new indications. First approved indications were significantly more likely to be of lower Phase design, e.g. Phase 1/2 rather than Phase 3. While this could mean that approvals are based on insufficient clinical data, further variables suggest otherwise. Initial approvals were also more likely to be shorter in duration and enroll more patients in the multivariate model. Therefore, initial drug approvals’ trials rely on a larger patient group, yet the expedited approval pathways permit (conditional) marketing authorization based on Phase 1 or 2 trials. Consequently, regulatory agencies and pharmaceutical companies prioritize indications providing a strong clinical benefit to patients with serious diseases. Clinical endpoint types did not significantly vary between initial and supplementary approvals, further suggesting that first indication approvals are not based on weaker clinical evidence.

The sequentially conducted multivariate regression analyses provide insights into the explanatory power of variable classes. Regulatory characteristics (Model 1) are the main factor that highlight differences in initial and supplementary indication approvals as illustrated by the Pseudo-R^2^ of 13.9%. Pivotal trial evidence (Model 2) further explains 5.8% and treatment characteristics (Model 3) 3.1% of the variation between initial and supplementary indication approvals. Nevertheless, the optimized Model 4 demonstrates that 77.6% of variation between characteristics of initial and supplementary indications remain unexplained. Whilst this observation could suggest that a large part of indication development is random, additional variables may be necessary to fully explain companies’ decision making in pharmaceutical development. For instance, variables characterizing an indication’s peak sales and competitive environment, the pharmaceutical company’s portfolio size, R&D partnerships of certain indications, licensing agreements, mergers and acquisitions, and underlying market conditions may influence economic decision of pharmaceutical companies throughout the drug development process [10, 11, 48].

Multi-indication cancer drugs may be classified into three categories according to indication similarity (presented in Sect. 2.1) [12 21, 25]. Results show that the initial and supplementary indication approval does not vary across this classification. In theory, it is expected that supplementary indication approvals for drugs used across therapeutic areas require additional evidence of Pre-clinical, Phase 1, 2, and 3 trials. In contrast, a single Phase 3 trial may be sufficient to extend a drug’s indication across different lines of therapy within the same disease. Future research with larger sample sizes should investigate whether clinical evidence, drug development timelines, and R&D costs differs across these different multi-indication cancer drugs.

### Strengths and limitations

This study relies on a uniquely large indication sample size covering all major regulatory agencies whilst analyzing several administrative and clinical variables. 25 targeted multi-indication drugs across 100 indications solidify the results of this study. Clinical and regulatory processes were analyzed in detail based on this longitudinal dataset collected from multiple data sources. The scope of collected variables permits an elaborate multivariate regression analysis which further verifies results by adjusting for multiple variables. To the best of our knowledge, this is the first study exploring the clinical evidence and regulatory approval of initial and supplementary indication approvals across four jurisdictions. Results offer important insights for government agencies, policy makers, and pharmaceutical companies.

Nevertheless, some limitations are present. First, conclusions drawn from this study are limited to oncology drugs. Second, the imputation of orphan status in Canada may positively impact the estimated odds ratio. Third, 18% of indications were combination treatments which could ultimately bias results. Combination treatments could potentially offer a higher clinical benefit relative to monotherapies, require higher Phase clinical trials, and are less frequently under priority review. Finally, restructurings and modernizations of regulatory approval processes during the investigation period limit conclusions. For instance, the TGA revised their orphan drug designation eligibility criteria in 2018.

Further research is necessary to investigate the effects of current pricing mechanisms on multi-indication drugs. A similar analysis of non-oncology multi-indication drugs is required to verify results in other treatment areas. The effect of pricing policies on the demand-side, e.g. usage and prescription, is of special interest for dispensers, prescribers, and patients [49].

## Conclusion

Targeted cancer drugs are increasingly approved across multiple indications. Regulatory agencies must evaluate these drugs’ safety and efficacy on an indication-specific level. We found that indication characteristics of multi-indication drugs significantly differ across regulatory agencies. Agencies employ expedited approval processes for similar indications to a different extent – more collaboration and transparency between regulatory agencies could create synergies for patients, regulatory agencies, and pharmaceutical companies. Results further suggest that both, regulatory agencies and pharmaceutical companies, prioritize and first approve orphan indications targeting serious diseases with a high unmet clinical need.

## Supplementary Information

Below is the link to the electronic supplementary material.

Supplementary file1 (PDF 291 KB)

Supplementary file1 (PDF 329 KB)

## Data Availability

All data used in this study were in the public domain. An example data extraction file is available from the corresponding author upon request.
