# Supplementary material for: Initial and supplementary indication approval of new targeted cancer drugs by the FDA, EMA, Health Canada, and TGA
Source: Invest New Drugs. 2022 Apr 7;40(4):798–809. doi: 10.1007/s10637-022-01227-5 (PMC9288371; doi:10.1007/s10637-022-01227-5)
Supplement: Supplementary file 1 — Supplementary file1 (PDF 291 KB) [file 10637_2022_1227_MOESM1_ESM.pdf]

Supplementary Online Content

Michaeli, D.T., Mill, M., Michaeli, T., Miracolo, A., Kanavos, P. Initial and supplementary indication approval of new targeted cancer drugs by the FDA, EMA, Health Canada, and TGA. *Investigational New Drugs* (2022). https://doi.org/10.1007/s10637-022-01227-5

Table e1 Marketing Authorization Data Sources

Table e2 Regulatory Approval Characteristics of Targeted Drugs Across the US (FDA), EU (EMA), Canada (HC), and Australia (TGA) – Continued

Table e3 Pearson Correlation Coefficients of Collected Variables

This supplementary material has been provided by the authors to give readers additional information about their work.

**Region**	**Agency**	**Website**
Europe	EMA	https://www.ema.europa.eu/en/medicines
Canada	HC	https://www.canada.ca/en/health-canada/services/drugs-health-products/drug-products/drug-product-database.html
Australia	TGA	https://www.tga.gov.au/ws-auspar-index
USA	FDA	https://www.accessdata.fda.gov/scripts/cder/daf/

Table e1 Marketing Authorization Data Sources

FDA: US Food and Drug Administration, EMA: European Medicines Agency, HC: Health Canada, TGA: Therapeutics Goods Administration.

	US (FDA)		EU (EMA)		Canada (HC)		Australia (TGA)		
	**No.**	**(%)**	**No.**	**(%)**	**No.**	**(%)**	**No.**	**(%)**	*P-Value*
**A) Regulatory**									
Approval Type									*<.001*
Standard	52	(54.2%)	74	(80.4%)	41	(47.7%)	72	(86.8%)	
Conditional Approval	28	(29.2%)	12	(13.1%)	23	(26.7%)	3	(3.6%)	
Priority Review	16	(16.6%)	6	(6.5%)	22	(25.6%)	8	(9.6%)	
Orphan Designation									*<.001*
No	49	(51.0%)	70	(76.1%)	NA	NA	75	(90.4%)	
Yes	47	(49.0%)	22	(23.9%)	NA	NA	8	(9.6%)	
MA Supporting Trial †									*<.001*
No	57	(59.4%)	25	(27.2%)	48	(55.8%)	50	(60.2%)	
Phase 1	7	(7.3%)	14	(15.2%)	5	(5.8%)	2	(2.4%)	
Phase 2	15	(15.6%)	28	(30.4%)	13	(15.1%)	19	(22.9%)	
Phase 3	17	(17.7%)	25	(27.2%)	20	(23.3%)	12	(14.5%)	
**B) Pivotal Trial**									
Trial Design ‡									*0.822*
Phase 1	4	(4.2%)	3	(3.2%)	5	(5.8%)	3	(3.6%)	
Phase 2	25	(26.0%)	18	(19.6%)	23	(26.8%)	19	(21.7%)	
Phase 3	67	(70.8%)	71	(77.2%)	58	(67.4%)	61	(74.7%)	
Primary Endpoint									*0.985*
Surrogate	63	(66.6%)	58	(63.0%)	60	(69.8%)	56	(67.5%)	
Clinical	17	(17.7%)	18	(19.6%)	14	(16.3%)	14	(16.9%)	
Co-Primary	16	(16.7%)	16	(17.4%)	12	(13.9%)	13	(15.6%)	
MCBS									*0.488*
Score of 1, 2, or 3	54	(56.2%)	55	(59.8%)	42	(48.8%)	48	(57.8%)	
Score of 4 or 5	42	(43.8%)	37	(40.2%)	44	(51.2%)	35	(42.2%)	
Enrolled Patients									
Mean	563	[479-647]	600	[525-685]	556	[463-648]	579	[485-673]	
Trial Length (months)									
Mean	33	[30-37]	30	[27-33]	34	[30-39]	32	[28-36]	
**C) Treatment**									
Treatment Type									*0.709*
Combination	17	(17.7%)	18	(19.6%)	12	(14.0%)	12	(14.5%)	
Monotherapy	79	(82.3%)	74	(80.4%)	74	(86.0%)	71	(85.5%)	
Line of Treatment									*0.959*
2nd, 3rd, 4th line	61	(63.5%)	55	(59.8%)	53	(62.6%)	52	(62.7%)	
1st line	35	(36.5%)	37	(40.2%)	33	(38.4%)	31	(37.3%)	
**Observations**	**96**	**(100%)**	**92**	**(100%)**	**86**	**(100%)**	**83**	**(100%)**	

Table e2 Regulatory Approval Characteristics of Targeted Drugs Across the US (FDA), EU (EMA), Canada (HC), and Australia (TGA) – Continued

P-values calculated based on χ^2-tests. FDA: US Food and Drug Administration, EMA: European Medicines Agency, HC: Health Canada, TGA: Therapeutics Goods Administration, MCBS: Magnitude of Clinical Benefit Scale (1: *low* benefit to 5: *high* benefit). † Highest Phase of *supporting* trials disclosed in the regulatory approval report (No supporting trial: 0, Phase 1: 1, Phase 2: 2, Phase 3: 3). ‡ Highest Phase *pivotal* trial disclosed in the regulatory approval report (Phase 1: 1, Phase 2: 2, Phase 3: 3).

		(1)	(2)	(3)	(4)	(5)	(6)	(7)	(8)	(9)	(10)	(11)	(12)
(1)	FDA Approval Sequence	1.00											
(2)	Conditional Approval	0.18***	1.00										
(3)	Priority Review	0.09	-0.20***	1.00									
(4)	Orphan Designation	0.21***	0.20***	0.13*	1.00								
(5)	Supporting Trials †	0.26***	0.07	-0.06	0.01	1.00							
(6)	Phase ‡	-0.18***	-0.48***	0.11*	-0.22***	0.03	1.00						
(7)	Primary Endpoint	-0.13*	-0.14**	-0.03	-0.31***	-0.01	0.34***	1.00					
(8)	MCBS Score	0.17**	0.32***	-0.10	0.29***	0.15**	-0.61***	-0.52***	1.00				
(9)	Enrolled Patients	-0.04	-0.23***	0.03	-0.35***	0.05	0.48***	0.46***	-0.36***	1.00			
(10)	Trial Length (months)	-0.12*	0.17***	-0.08	-0.01	-0.09	-0.40***	-0.12*	0.18***	0.03	1.00		
(11)	Monotherapy	0.20***	0.08	0.06	0.21***	0.08	-0.26***	-0.51***	0.37***	-0.38***	0.01	1.00	
(12)	1st Line Treatment	-0.11*	-0.23***	0.04	-0.12*	0.03	0.27***	0.10	-0.12*	0.11*	-0.03	-0.35***	1.00

Table e3 Pearson Correlation Coefficients of Collected Variables

Indication approval sequence was determined by the FDA approval date (initial indication: 1, supplementary indication: 0). P-values: * *p<0.05*, ** *p<0.01*, *** *p<0.001*. MCBS: Magnitude of Clinical Benefit Scale (1: *low* benefit to 5: *high* benefit). † Highest Phase of supporting trials disclosed in the regulatory approval report (No supporting trial: 0, Phase 1: 1, Phase 2: 2, Phase 3: 3). ‡ Highest Phase pivotal trial disclosed in the regulatory approval report (Phase 1: 1, Phase 2: 2, Phase 3: 3).
