# Supplementary material for: Initial and supplementary indication approval of new targeted cancer drugs by the FDA, EMA, Health Canada, and TGA
Source: Invest New Drugs. 2022 Apr 7;40(4):798–809. doi: 10.1007/s10637-022-01227-5 (PMC9288371; doi:10.1007/s10637-022-01227-5)
Supplement: Supplementary file 2 — Supplementary file1 (PDF 329 KB) [file 10637_2022_1227_MOESM2_ESM.pdf]

Supplementary Table 1: QGRS/PQS sequences in SARS-CoV2 genome with overlap (forward strand)

QGRS/PQS sequences found (overlaps included) (Forward strand)

Position	Length	QGRS/PQS	G-Score
353	22	GGCTTTGGAGACTCCGTGGAGG	13
353	25	**GGCTTTGGAGACTCCGTGGAGGAGG**	16
353	25	GGCTTTGGAGACTCCGTGGAGGAGG	10
353	25	GGCTTTGGAGACTCCGTGGAGGAGG	7
359	19	GGAGACTCCGTGGAGGAGG	13
370	20	GGAGGAGGTCTTATCAGAGG	12
644	20	**GGTAATAAAGGAGCTGGTGG**	15
653	23	GGAGCTGGTGGCCATAGTTACGG	12
1462	27	GGGTGGTCGCACTATTGCCTTTGGAGG	6
1463	26	**GGTGGTCGCACTATTGCCTTTGGAGG**	6
1574	26	**GGTGTTGTTGGAGAAGGTTCCGAAGG**	18
2714	29	**GGCGGTGCACCAACAAAGGTTACTTTTGG**	10
3467	17	**GGAGGAGGTGTTGCAGG**	15
3470	28	GGAGGTGTTGCAGGAGCCTTAAATAAGG	10
4162	27	**GGTTATACCTACTAAAAAGGCTGGTGG**	6
4261	29	**GGGTTTAAATGGTTACACTGTAGAGGAGG**	10
4262	28	GGTTTAAATGGTTACACTGTAGAGGAGG	10
8687	23	**GGATACAAGGCTATTGATGGTGG**	14
10255	28	GGTACAGGCTGGTAATGTTCAACTCAGG	9
10255	29	GGTACAGGCTGGTAATGTTCAACTCAGGG	8
10261	30	**GGCTGGTAATGTTCAACTCAGGGTTATTGG**	9

10261	30	GGCTGGTAATGTTCAACTCAGGGTTATTGG	8
13385	20	**GGTATGTGGAAAGGTTATGG**	19
14947	28	**GGTTTTCCATTTAATAAATGGGGTAAGG**	4
15208	27	**GGAACAAGCAAATTCTATGGTGGTTGG**	6
15448	29	**GGCGGTTCACTATATGTTAAACCAGGTGG**	3
18296	23	**GGATTGGCTTCGATGTCGAGGGG**	9
22316	29	**GGTGATTCTTCTTCAGGTTGGACAGCTGG**	10
24199	30	GGGTACAATCACTTCTGGTTGGACCTTTGG	9
24200	29	GGTACAATCACTTCTGGTTGGACCTTTGG	10
24215	20	**GGTTGGACCTTTGGTGCAGG**	17
24268	24	**GGCTTATAGGTTTAATGGTATTGG**	19
25197	22	**GGCCATGGTACATTTGGCTAGG**	17
25203	28	GGTACATTTGGCTAGGTTTTATAGCTGG	14
25951	29	**GGTGGTTATACTGAAAAATGGGAATCTGG**	8
25951	29	GGTGGTTATACTGAAAAATGGGAATCTGG	7
26746	30	**GGATCACCGGTGGAATTGCTATCGCAATGG**	7
28781	29	**GGCTTCTACGCAGAAGGGAGCAGAGGCGG**	9
28781	29	GGCTTCTACGCAGAAGGGAGCAGAGGCGG	8
28881	28	GGGGAACTTCTCCTGCTAGAATGGCTGG	3
28903	15	**GGCTGGCAATGGCGG**	18
29123	19	**GGAAATTTTGGGGACCAGG**	14
29132	26	GGGGACCAGGAACTAATCAGACAAGG	7
29234	30	**GGCATGGAAGTCACACCTTCGGGAACGTGG**	11
29234	30	GGCATGGAAGTCACACCTTCGGGAACGTGG	10

Supplementary Table 2: QGRS/PQS sequences in SARS-CoV2 genome with overlap (reverse strand)

QGRS/PQS sequences found (overlaps included) (Reverse strand)

Position	Length	QGRS/PQS	G-Score
165	22	**GGCCTCGGTGAAAATGTGGTGG**	13
2987	29	**GGTCTGGTCAGAATAGTGCCATGGAGTGG**	9
6822	30	**GGTTGGTAACCAACACCATTAGTGGGTTGG**	6
6822	30	GGTTGGTAACCAACACCATTAGTGGGTTGG	5
11420	28	GGTGTTTAAATTGATCTCCAGGCGGTGG	4
11440	25	**GGCGGTGGTTTAGCACTAACTCTGG**	7
13136	19	**GGTTAAGTGGTGGTCTAGG**	16
13944	27	GGCCCCTAGGATTCTTGATGGATCTGG	16
13944	28	GGCCCCTAGGATTCTTGATGGATCTGGG	17
13952	25	GGATTCTTGATGGATCTGGGTAAGG	16
13952	25	GGATTCTTGATGGATCTGGGTAAGG	15
13952	29	GGATTCTTGATGGATCTGGGTAAGGAAGG	16
13952	29	GGATTCTTGATGGATCTGGGTAAGGAAGG	17
13952	29	GGATTCTTGATGGATCTGGGTAAGGAAGG	13
13952	29	GGATTCTTGATGGATCTGGGTAAGGAAGG	8
13952	29	GGATTCTTGATGGATCTGGGTAAGGAAGG	7
13963	18	**GGATCTGGGTAAGGAAGG**	19
13963	18	GGATCTGGGTAAGGAAGG	18
16623	27	**GGATTTGGATGATCTATGTGGCAACGG**	14
19865	21	GGTGATAGAGGTTTGTGGTGG	15

19865	25	**GGTGATAGAGGTTTGTGGTGGTTGG**	19
19865	25	GGTGATAGAGGTTTGTGGTGGTTGG	15
19865	25	GGTGATAGAGGTTTGTGGTGGTTGG	8
19874	16	GGTTTGTGGTGGTTGG	17
23877	17	**GGATATGGTTGGTTTGG**	19
23883	26	GGTTGGTTTGGTACAAGATCAATTGG	10
25003	16	**GGTGGAATGTGGTAGG**	17
27432	24	**GGGGCTTTTAGAGGCATGAGTAGG**	13
29867	23	**GGTTGGTTTGTTACCTGGGAAGG**	13
29867	23	GGTTGGTTTGTTACCTGGGAAGG	12
